# Long-term outcome after routine surgery for pelvic organ prolapse—A national register-based cohort study

**DOI:** 10.1007/s00192-022-05156-y

**Published:** 2022-03-21

**Authors:** Emil Nüssler, Gabriel Granåsen, Marie Bixo, Mats Löfgren

**Affiliations:** Department of Clinical Sciences, Obstetrics and Gynaecology, Umeå University, 901 85 Umeå, Sweden

**Keywords:** Pelvic organ prolapse, Kaplan-Meier estimates, Follow-up studies, Population register, Surveys and questionnaires

## Abstract

**Introduction and hypothesis:**

Pelvic organ prolapse (POP) is common, and women have an estimated 12–19% lifetime risk for needing POP surgery. Aims were to measure re-operation rates up to 10 years after POP surgery and patient-reported outcomes (PROMs) 5 years after a first-time operation for POP.

**Methods:**

This is a cohort study using the Swedish National Quality Register for Gynaecological Surgery (GynOp). We retrieved information from 32,086 POP-operated women up to 10 years later. After validation, a web-based PROM questionnaire was sent to 4380 women who 5 years previously had standard POP surgery. Main outcome measures were reoperations due to a relapse of prolapse and PROMs 5 years after the primary operation.

**Results:**

Among women operated for all types of POP, 11% had re-operations 5 years later and an additional 4% 10 years later, with similar frequencies for various compartments/types of surgery. PROMs yielded a 75% response rate after 5 years. Cure rate was 68% for anterior, 70% for posterior, and 74% for combined anterior-posterior native repairs. Patient satisfaction exceeded 70%, and symptom reduction was still significant after 5 years (*p* < 0.0001).

**Conclusions:**

Following primary prolapse surgery, re-operation rates are low, even after 10 years. A web-based survey for follow-up of PROMs after POP surgery is feasible and yields a high response rate after 5 years. The subjective cure rate after primary POP operations is high, with reduced symptoms and satisfied patients regardless of compartment. Standard prolapse surgery with native tissue repair produces satisfactory long-term results.

**Supplementary Information:**

The online version contains supplementary material available at 10.1007/s00192-022-05156-y.

## Introduction

Pelvic organ prolapse (POP) is common worldwide, with women having a 12–19% lifetime risk of needing surgery for this condition [1, 2]. All surgical procedures for POP since 2006 have been registered in the Swedish National Quality Register for Gynaecological Surgery (GynOp), which has national coverage [3]. The most common sites needing repair are the anterior and posterior vaginal compartments [3]. The mean age of Swedish women operated on for POP is 60.3 years, and because the average expected lifespan for women in Sweden is 84, these women are expected to live another 24 years [4] after the operation. POP is not a life-threatening condition, but it does have a negative impact on quality of life [5, 6]. The condition is often neglected, and more research on the long-term outcome of POP surgery is warranted.

Data on both patient-reported operative results and re-treatment rates have varied widely in previous studies. One study reported a re-operation rate of 29.2% [7]. Another recent study estimated that the risk of relapse was 15.4%, based on subjective symptoms of bulging, and 21.2%, based on clinical examination (mean time of follow-up was 47 months) [8]. A study of surgery for severe prolapse reported a 12.8–36.2% relapse at the clinical follow-up examination after 12 months [9]. Overall, results are conflicting regarding the long-term results of native tissue repair and highly heterogenous when it comes to outcome measures or defining a successful or failed operation, as has been shown in several Cochrane reviews [10, 11]. In addition, studies with follow-up times of > 1 or 2 years for larger populations have mainly focused on comparing techniques, not on the durability of the surgical repair per se [10, 11]. No studies of the combined effects on patient-reported outcomes (PROMs) and operative results in the same population more than a year after the operation have been published [12–16]. Assessing PROMs should give us an overview of the patients’ various problems related to POP, including the effects of surgery on the typical symptoms, and should relate solely to the original primary operation regardless of whether subsequent re-operations were performed or not. Another way to look at long-term results is to evaluate the re-operation rates, which we believe will reflect the relapses with the most severe symptoms. In this study, we have chosen to look at both measures. The underlying rationale for our study was to evaluate the long-term results of routine surgical techniques for treating the most common types of POP in a large, unselected population from a quality register with national coverage, and to base this evaluation on both PROMs and objective outcomes (re-operations).

### Aims

- To measure the frequency of re-operations at 5 and 10 years after an original operation for POP;
- To test the feasibility and validity of a web-based questionnaire for doing a follow-up PROM 5 years after surgery for POP
- To use the validated questionnaire for a follow-up PROM for women who had undergone native tissue repair for primary POP 5 years ago.

## Materials and methods

### The Swedish national quality register for gynaecological surgery

The GynOp register was established in 1997, and since 2005 it has collected information about prolapse surgery. By 2009, 33 out of 55 hospitals nationwide had started to register POP surgery in GynOp, and by 2017, all Swedish hospitals were participating. A comparison between GynOp and the National Patient Register, which is administered by the National Board of Health and Welfare, shows that GynOp covers 3% more patients on average per year than the obligatory National Patient Register. The coverage of GynOp is estimated to be 90%, and a recent matching (2010–2017) between the two registers showed 77% correspondence between procedures in the two registers [13, 17].

### Validation of register data

For this study, information about re-operations after surgery for POP was extracted directly from the GynOp register. A total of 41,828 patients were registered as having undergone surgery in the period from 2 February 2005 to 31 December 2017. Utilizing the preoperative health assessments and declarations by both the physicians and the patients, we cross-tabulated the answers in the questionnaires. Cross-tabulation of patients’ and surgeons’ answers (*n* = 4629) where a previous operation was already registered showed that if either the patient or the physician indicated that a previous operation had been done, they were correct in 97% of cases. Almost none (0.5%) of the patients lacked information about previous operations (Table 1).

**Table 1:** Cross-tabulation of patients’ and surgeons’ answers to the question “Have you/has the patient had a previous POP operation?” (*n* = 4629)

		Has the surgeon reported a previous POP operation?^a^				Total
		No patient history available	Confirmed a previous POP operation	Dismissed a previous POP operation^f^	Did not answer^c^	
Has the patient reported a previous POP operation?^b^	Did not answer^c^	24^e^	534	29	12^e^	599
	Confirmed a previous gynaecological operation	7	376	44	11	438
	Rejected a previous POP operation	2	48	5	0	55
	No previous answers registered in GynOp	0	85	3	2	90
	Confirmed a previous POP operation	38	3215	138^f^	56	3,447
Total		71	4258	219	81	4,629^d^

GynOp = Swedish National Quality Register for Gynaecological Surgery; POP = pelvic organ prolapse

^a^Surgeons’ answers (4629–81 = 4548) regarding previous operation (4258) yielded a correct classification of patients (by the surgeons alone) of 93%

^b^The patients’ answers (4629−599 = 4030) regarding previous operations (3447) yielded a correct classification of patients (by the patients alone) of 85.6%

^c^Patients answered the question in 4030 of 4629 cases (87.1%)

^d^In total, the patients and surgeons answered the question correctly in 97% of cases

^e^In 0.8% of cases, neither the patient nor the surgeon answered the question

^f^Doctors dismissed a previous POP operation in 5.8% of cases. Of those, 138 (63%) were reported by the patients as recurrent operations (i.e., as previous POP operation)

To create the study database, including only natively operated primary patients, the cross-tabulation results were used to exclude patients with previous POP operations. Thus, out of the original 41,828 patients, 8294 (19.8%) were excluded because the candidate surgery was not their first POP surgery. An additional 1448 (3.4%) who were operated on using surgical mesh in the anterior or posterior compartment were excluded to avoid case mix. Excluding these patients left 32,086 women who had undergone a first-time POP operation, and these became our study group for the survival curve analyses.

### Validation and design of the patient-reported outcomes questionnaire

The 5-year questionnaire consisted of previously validated questions [18, 19] used routinely in GynOp (preoperatively and at 1-year follow-up) and a set of new questions. In total, 29 questions about patient-reported general health, symptoms, and outcomes, including a feeling of genital protrusion, were taken from the previous questionnaires. In addition, nine new questions regarding details about mesh operations or designed to assess patient-reported re-operations were added to this 5-year questionnaire (the results of these questions are not presented in this study). All questions were phrased to require either dichotomous answers (yes/no) or one of five possible answers (a scale ranging from ‘never’ to ‘daily symptoms’).

The study group for validation of this 5-year questionnaire comprised 40 patients who had been operated on 5 years previously and were selected at random from the GynOp database. Interviews were conducted in two rounds of 20 patients each. The patients were contacted by mail and asked if they would agree to a telephone interview with a researcher from GynOp regarding a new questionnaire. The in-depth interviews were done to face-validate the questionnaire, and all questions were scrutinized individually by the patients. These interviews took about 40 min. The validation procedure is summarised in Supplemental Fig. 5.

### Data from the patient-reported outcomes questionnaire

To ensure that the patient-reported results of this study reflected the original native operations and yielded clinically relevant results, we selected the following groups for the PROMs analysis:

- patients operated with native tissue repair solely for cystocele;
- patients operated with native tissue repair solely for rectocele;
- patients who had undergone simultaneous rectocele and cystocele operations

Patients surgically re-treated for POP at any point during the 5-year follow-up period and patients with any concomitant surgery, including incontinence procedures and apical suspension, were excluded.

### Statistical methods

Survival functions for the time to re-treatment were estimated using Kaplan-Meier curves. Observations were censored if no re-treatment had occurred by the time of data extraction from the registry. Differences in baseline characteristics were analysed using the Student’s *t*-test for continuous outcomes and the chi-square test for proportions. The chi-square test was used to compare preoperative symptoms and symptoms still present after 5 years. Statistical analyses were performed using R (R v3.5.3, R Core Team, Vienna, Austria) and SPSS Statistics for Windows, version 24.0 (IBM Corp, Armonk, NY, USA).

### Ethics approval

The register has an overall approval from the Regional Ethical Review Board in Umeå (Dnr 04-107, 27 May 2005). For the present study a specific approval was granted (Dnr 08-076M 2 September 2018). All patients contributing information to GynOp have the opportunity to be excluded from the register according to Swedish law.

## Results

### Survival curves (for re-treatment) of different pelvic organ prolapse procedures

Baseline characteristics of the 32,086 women in the original cohort who had undergone one primary prolapse operation and thereafter been included in the survival analyses for re-operation are shown in Table 2. The Kaplan-Meier curves cover up to 12 years postoperatively. All POP operations were performed using native tissue repair except the vaginal apex operations (see below).

**Table 2:** Baseline characteristics of the study cohort (*n* = 32,086) at the time of the original primary operation for pelvic organ prolapse (POP)

	Cystocele (*n* = 13,809)	*n*	Missing (%)	Rectocele (*n* = 5846)	*n*	Missing (%)	Vaginal apex (*n* = 4533)	*n*	Missing (%)	Remaining types^a^ (*n* = 7898)	*n*	Missing (%)
Age (mean years, 95% CI)	64.8 (64.8–64.9)	13,809	0	57.1 (56.8–57.5)	5,846	0	63.5 (63.2–63.8)	4,533	0	61.1 (60.8–61.4)	7,898	0
ASA score (mean, 95% CI)	1.53 (1.52–1.54)	13,525	1.5	1.48 (1.47–1.49)	5,770	1.3	1.59 (1.57–1.61)	4,486	0.66	1.50 (1.49–1.51)	7,737	1.6
Parity (mean *n*, 95% CI)	2.45 (2.43–2.46)	12,156	11.3	2.52 (2.49–2.55)	5,026	13.3	2.52 (2.49–2.55)	4,041	10.3	2.53 (2.51–2.55)	6,837	12.8
BMI (95% CI)	26.0 (25.9–26.1)	12,004	12.7	26.5 (26.4–26.6)	5,008	14.3	25.8 (25.7–25.9)	3,994	11.9	26.2 (26.1–26.3)	6,770	14.3
Active smokers (%, 95% CI)	9.2 (8.70–9.70)	12,619	8.6	9.4 (8.6–10.2)	5,181	11.4	9.4 (8.5–10.3)	4,156	8.3	8.8 (8.1–9.5)	7,070	10.5
Chronic coughing or asthma (%, 95% CI)	15.7 % (15.1–16.3)	12,584	8.9	20.3 (19.2–21.4)	5,159	11.8	15.7 (14.6–16.8)	4,137	8.7	17.6 (16.7–18.5)	7,044	10.8
Currently employed (%, 95% CI)	42.0 (41.1–42.9)	11,136	19.4	56.0 (54.6–57.4)	4,838	17.2	44.0 (42.4–45.6)	3,906	13.8	51.0 (48.9–52.2)	6,370	19.3
Pensioners (%, 95% CI)	43.0 (42.1–43.9)	11,136	19.4	24.0 (22.8–25.2)	4,838	17.2	44.0 (42.4–45.6)	3,906	13.8	36.0 (34.8–37.2)	6,370	19.3
Had performed heavy physical labour (%, 95% CI)	18.0 (17.3–18.7)	12,834	7.1	21.0 (19.9–22.1)	5,262	10.0	21.0% (19.8–22.2)	4,185	7.7	22.0 (21.0–23.0)	7,203	8.7
Operation time (minutes, 95% CI)	45 (45–46)	12,288	11.0	47 (46–48)	5,300	9.3	81 (80–82)	4,283	5.5	68 (67–68)	7,117	9.9

^a^All other types of prolapse surgery, including combinations of cystocele, rectocele, and vaginal apex operations

ASA = American Society of Anesthesiologists; BMI = body mass index; 95% CI = 95% confidence interval

### All POP operations regardless of compartment (*n* = 32,086)

**Fig. 1:**
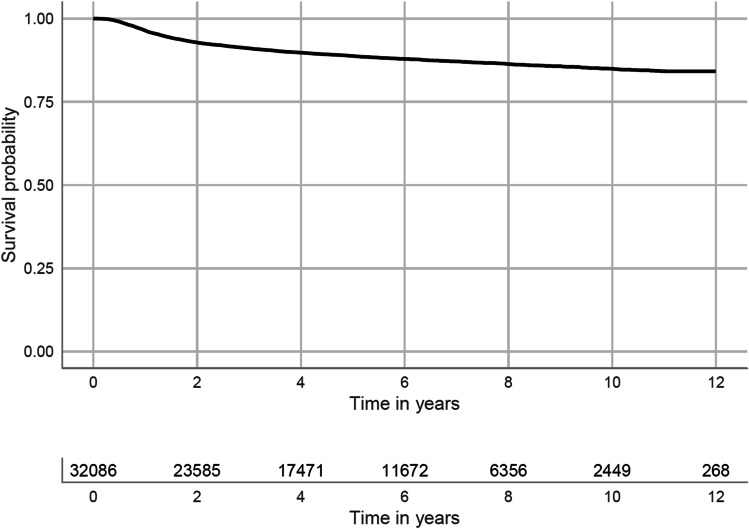
Kaplan-Meier estimate of all primary POP-operated patients (*n* = 32,086). The line underneath the curve shows the risk of retreatment up to 12 years after all primary POP operations pooled together, regardless of compartment

Figure 1 shows the survival curve for re-operations after all kinds of primary POP operations pooled together. Overall, 11.2% of the patients had undergone a re-operation 5 years later and 15% had undergone re-operation 10 years later.

### Primary operations in the anterior compartment (*n* = 13,809)

**Fig. 2:**
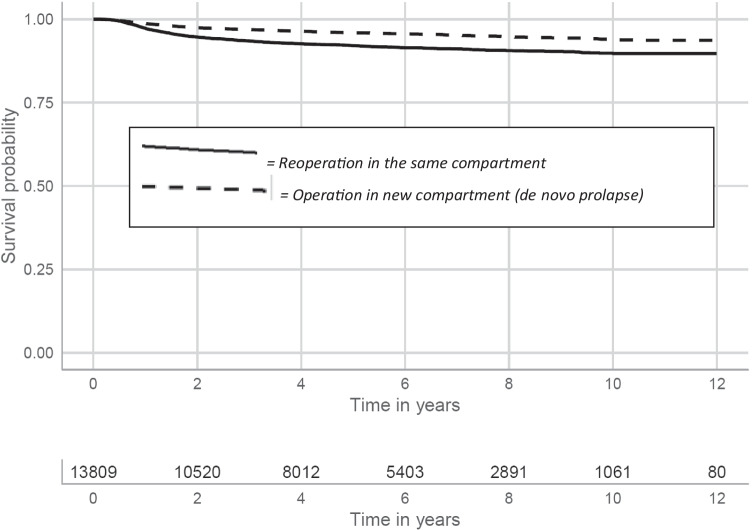
Kaplan-Meier estimates for cystocele primary operated patients (*n* = 13,809) with native tissue repair. The curves have been split to show the risk of relapse in the same compartment and the risk of de novo prolapse in a different vaginal compartment

After a primary operation solely in the anterior compartment, 11.7% and 15.7% of the patients had re-operations 5 and 10 years later, respectively. A stratification based on the site of re-treatment revealed that most re-operations were performed in the same (anterior) compartment. Altogether, 7.9% (5 years later) and 10.3% (10 years later) had re-operations for a relapse in the anterior compartment. The frequencies for prolapse in a different compartment were 4.1% and 6.1% at 5 and 10 years later, respectively (Fig. 2).

### Primary operations in the posterior compartment (*p* = 5846)

**Fig. 3:**
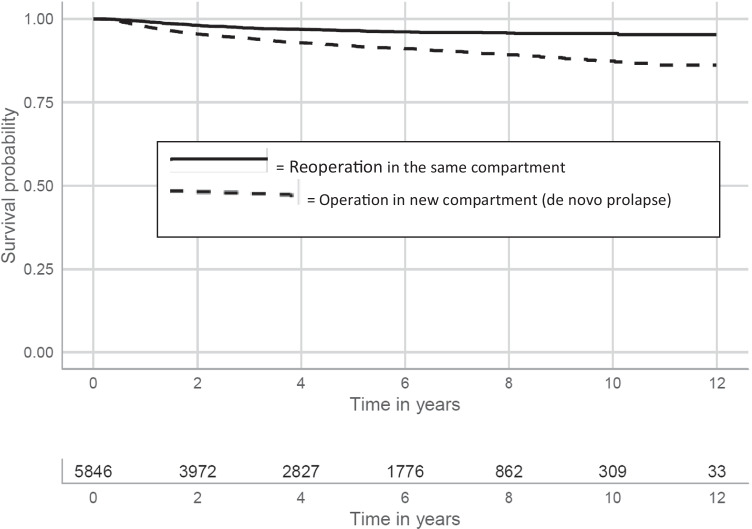
Kaplan-Meier estimates for solely rectocele operated patients (*n* = 5846) with native tissue repair. The curves have been split to show the risk of relapse in the same compartment and the risk of de novo prolapse in a different vaginal compartment

After a primary operation solely in the posterior compartment, 11.5% and 16.4% of the patients had re-operations 5 and 10 years later, respectively. In contrast to the group described above, the most common site for re-treatment was a different compartment, at 8.0% 5 years later and 12.6% 10 years later. The frequencies for re-operations in the posterior compartment were 3.5% 5 years later and 4.4% 10 years later (Fig. 3).

### Combined anterior and posterior repair, with or without concurrent cervix surgery (*n* = 5502)

A combination of both posterior and anterior native tissue repair, with or without concurrent cervix surgery (cervical amputation), is considered the “classical” Manchester procedure. The frequencies of re-operations in these cases were 9.0% and 11.2% after 5 and 10 years, respectively.

### Primary vaginal apex operations (*n* = 4533)

There are different procedures for vaginal apex operations including open, laparoscopic or vaginal approaches, with or without mesh and various fixations to anatomical structures. All operations in this group were solely on the vaginal apex and with no concurrent surgery of any kind, e.g., cysto- or rectocele. All vaginal apex operations were pooled into one group. The risk of re-treatment after a vaginal apex operation was 12.9% 5 years later and 17% 10 years later.

### All other primary vaginal POP operations, including concurrent surgery (*n* = 2396)

After excluding the groups above, a group of 2396 patients remained. None of these women had been operated on using surgical mesh or other implants.

The group consisted of women who had undergone any of the procedures below:

- operation for a cystocele, combined with either incontinence or enterocele surgery, *n* = 288 (12%);
- operation for a rectocele, combined with either incontinence or enterocele surgery, *n* = 1103 (46%;
- kolpokleisis, *n* = 216 (9%);
- total hysterectomy (in 41 cases combined with an enterocele operation), *n* = 522 (22%);
- unclassified operation, *n* = 267 (11%).

Taken together, 10.6% and 14.4% of the patients in this group had re-operations 5 and 10 years later, respectively.

### Validation of the 5-year follow-up questionnaire

During the first validation round, 13 out of 20 patients completed the telephone interview, after which five changes were made to the nine new mesh-specific questions. These revisions to the original questionnaire warranted another round of validation through patient interviews, so 20 additional patients were contacted and interviewed. These interviews showed that only minor rephrasings were needed, but the introduction letter was also shortened. During both validation rounds, none of the questions in the preoperative or 1-year questionnaires were criticized.

### Feasibility of the 5-year follow-up questionnaire

**Fig. 4:**
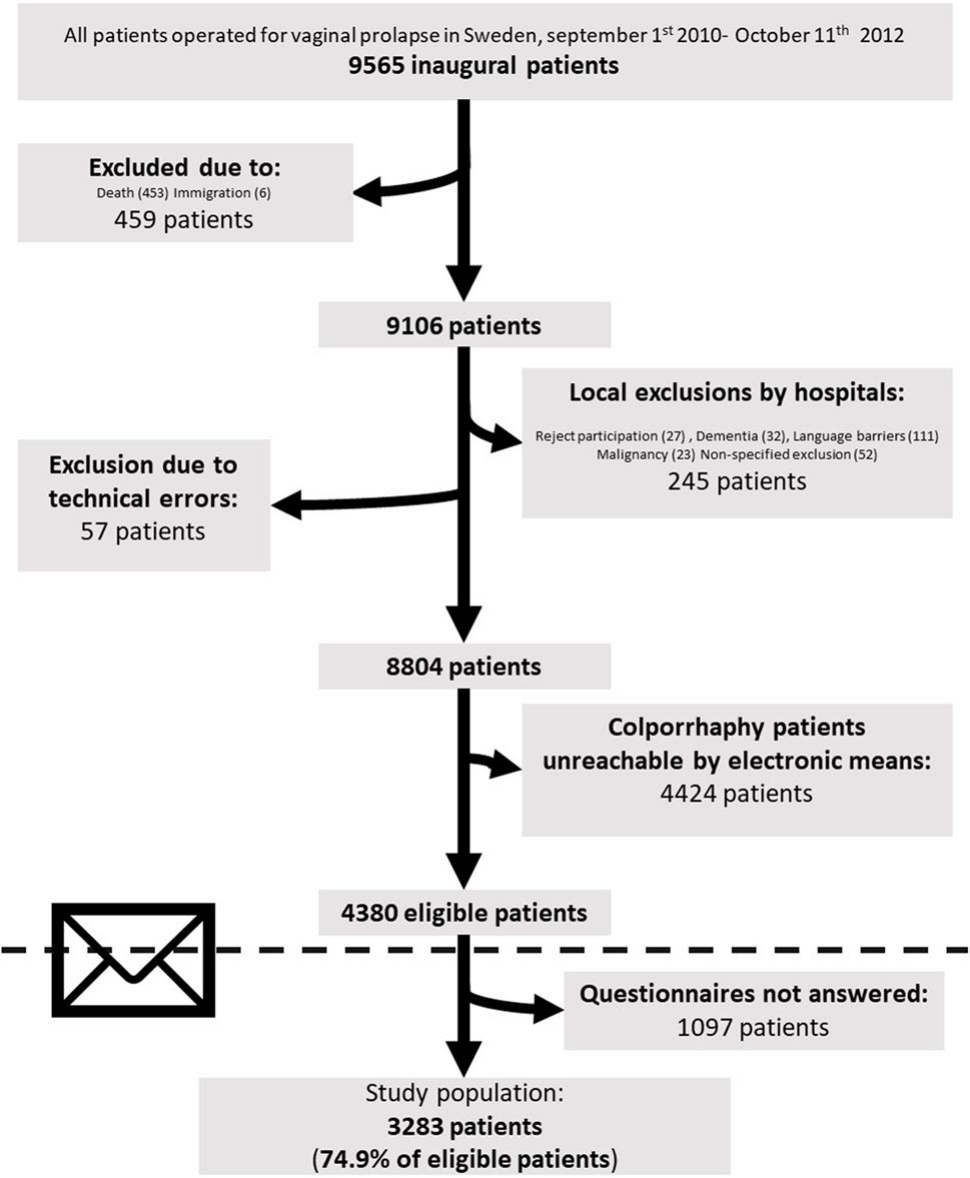
Flow chart showing selection of participants for the Patient Reported Outcome Measures (PROM) after 5-year follow-up (*n* = 3283)

To find all patients eligible to answer the questionnaire, we identified all patients registered in GynOp who had been operated on at least 5 years previously but at most 5.9 years previously (between 10 October 2010 and 10 September 2011). After determining this initial study group, we continuously identified and followed up with patients who had been operated on 5 years previously up until 11 October 2012. A total of 9565 eligible patients were identified. Patients were excluded by the local hospital if they were dead, lacked e-mail addresses, or were otherwise unsuitable (Fig. 4). The excluded patients were similar in all background parameters to the patients included in the study except for the lack of an e-mail address, although they were slightly older (2 years). The final population eligible for follow-up consisted of 4380 patients.

Questionnaires were first sent out via e-mail, and, if these were not responded to, a reminder was sent in the mail 2 weeks later. If patients did not respond after the reminder, a paper questionnaire was sent by mail. Response rates were similar whether patients were given electronic questionnaires or paper questionnaires (the latter were given to mesh-operated patients who lacked an e-mail address) at 74.9% and 76.4%, respectively. The response rate of all completed questionnaires by natively operated patients was 74.9%, resulting in a study population of 3283 patients (Fig. 4).

### Patient-reported outcome measurements after 5 years

#### Patient characteristics and drop-out analysis

A total of 1097 patients did not answer the questionnaire (Fig. 4). Drop-out analysis revealed that compliant patients were slightly older than non-compliant patients (mean ages of 60.9 versus 58.9 respectively at the time of operation) and were less likely to be active smokers (7. 2% versus 12.2% respectively at the time of operation). No other significant differences were found, including body mass index (BMI), parity, current employment status, hard physical labour, chronic coughing, physical health status, or any other comorbidities.

#### Primary cystocele patients, main outcome (*n* = 928)

The patient-reported cure rate (absence of a feeling of vaginal bulging) 5 years after this operation was 68.2%. Most patients (75.5%) reported being satisfied or very satisfied with the results of the operation, and there was a high (83.1%) patient-reported rate of improvement of symptoms compared to their preoperative condition (Table 3 lists the symptoms, including urinary symptoms, defecation symptoms, sexual activity, and vaginal chafing).

**Table 3:** Patient-reported outcomes (PROMs) 5 years after a primary operation for pelvic organ prolapse (POP) with native tissue repair (*n* = 1782). The Table reports PROMs for women operated for cystocele, rectocele, or a simultaneous cystocele and rectocele operation

PROMs (%, 95% CI)	Cystocele (*n* = 928)	*n*	Missing *n* (%)	*n*	Missing *n* (%)	Rectocele (*n* = 410)	Combination (*n* = 444)	*n*	Missing *n* (%)
Cure rate (%, 95% CI)	68.2 (65.2–71.2)	917	11 (1.2)	70.2 (65.7–74.7)	404	6 (1.5)	74.4 (70.3–78.5)	441	3 (0.7)
Satisfied overall with the operation (%, 95% CI)	75.5 (72.7–78.3)	924	4 (0.4)	70.2 (65.8–74.6)	408	2 (0.5)	73.2 (69.1–77.4)	437	7 (1.6)
Symptoms have improved (%, 95% CI)	83.1 (80.7–85.5)	924	4 (0.4)	78.3 (74.3–82.3)	409	1 (0.2)	82.2 (78.6–85.8)	438	6 (1.4)
Vaginal chafing (%, 95% CI)	12.8 (10.6–14.9)	919	9 (1.0)	18.7 (14.9–22.5)	406	4 (1.0)	13.1 (10.0–16.3)	440	4 (0.9)
	3.2 (2.0–4.3)^a^			5.6 (3.4–7.8)^a^			2.9 (1.3–4.5)^a^		
Voiding difficulties (%, 95% CI)	28.7 (25.8–31.6)	918	10 (1.1)	33.6 (29.0–38.2)	406	4 (1.0)	60.4 (55.8–64.9)	438	6 (1.4)
	11.6 (9.5–13.7)^a^			12.7 (9.5–15.9)^a^			12.2 (9.13–15.3)^a^		
Urinary incontinence (%, 95% CI)	32.1 (29.1–35.1)	919	9 (1.0)	44.4 (39.6–49.2)	407	3 (0.7)	34.7 (30.3–39.2)	440	4 (0.9)
	9.3 (7.4–11.2)^a^			12.9 (9.6–16.2)^a^			8.8 (6.2–11.5)^a^		
Bladder urgency (%, 95% CI)	48.5 (45.3–51.7)	917	11 (1.2)	55.1 (50.3–59.9)	407	3 (0.7)	52.4 (47.7–57.0)	441	3 (0.7)
	20.0 (17.4–22.6)^a^			22.0 (18.0–26.0)^a^			20.0 (16.3–23.7)^a^		
Defecation problems (%, 95% CI)	29.8 (26.8–32.8)	915	13 (1.4)	50.7 (45.8–55.6)	406	4 (1.0)	39.2 (34.6–43.8)	440	4 (0.9)
	4.6 (3.2–5.9)^a^			13.4 (10.1–16.7)^a^			7.7 (5.2–10.2)^a^		
Sexually active (%, 95% CI)	43.4 (40.2–46.6)	915	13 (1.4)	56.3 (51.5–61.1)	407	3 (0.7)	52.0 (47.3–56.7)	440	4 (0.9)
Dyspareunia (%, 95% CI) ^b^	10.7 (7.7–13.7) 2.6 (1.0–4.7)^c^	401	2 (0.5)	21.3 (16.0–26.6)	231	0	18.1 (13.1–23.1)	229	10 (0.9)
				8.6 (5.0–12.22)^c^			0.9 (0–2.1)^c^		

Several PROM subgroups are noted here to give a complete perspective on the functional parameters of the women involved, especially regarding daily or severe symptoms of defecation problems or dyspareunia

^a^Subgroup reporting daily symptoms

^b^Subgroup of sexually active women

^c^Subgroup with moderate to severe dyspareunia

95% CI = 95% confidence interval

#### Primary rectocele patients, main outcome (*n* = 410)

The patient-reported cure rate 5 years after this operation was 70.2%. Most patients (70.5%) reported being satisfied or very satisfied with the results of the operation and there was a high (78.3%) patient-reported rate of improvement of symptoms compared to their preoperative condition (Table 3 lists the secondary parameters of urinary symptoms, defecation symptoms, reduced sexual activity, and vaginal chafing).

#### Primary patients operated with native tissue repair for a combination of anterior and posterior prolapse, main outcome (*n* = 444)

The patient-reported cure rate 5 years after this operation was 74.4%. Most patients (73.2%) reported being satisfied or very satisfied with the results. Improvement of symptoms was 82.2% (Table 3 lists the secondary parameters).

#### Patient-reported outcomes for all groups

Urinary and/or defecation problems varied among the three groups, in both intensity and the proportion of patients who experienced them (Table 3). To evaluate the long-term effects on these symptoms by type of prolapse surgery, a comparison between the PROMs in the preoperative patient questionnaire and the 5-year questionnaire was performed. This analysis revealed that:

- urinary symptoms were significantly reduced after a cystocele operation (*p* < 0.001);
- defecation problems were significantly reduced after a rectocele operation (*p* < 0.001);
- sexual activity was reduced regardless of the type of operation;
- for the subgroup of 4.8% (*n* = 157) of patients who answered preoperatively that they had no genital protrusion, there was no change regarding the feeling of a vaginal bulge;
- symptoms of vaginal bulging preoperatively were significantly reduced 5 years after the operation (*p* < 0.001).

## Discussion

In this study, we found that there was a low proportion of re-treatments after primary surgery for POP: < 12% had been re-operated 5 years later and < 15% 10 years later. This figure is lower than in many other studies, which have reported re-treatment rates and/or relapses between 22% and 33% after 1 year [20–22]. In part, the difference in results may be due to discrepancies in diagnosing and defining relapse of prolapse. It should be noted that our 5-year PROM results show that approximately 30% of the patients had symptoms related to prolapse but these did not prompt a reoperation.

All data in this study were prospectively collected in a national register reflecting routine healthcare. Only the research question of reoperation was retrospectively constructed from this prospectively collected data.

A strength of this study is that the population captured was large enough to permit stratification into different compartments and different procedures and to distinguish between “re-treatments” occurring in the same compartment (a recurrence) or in a different compartment (de novo). Most retreatments after rectocele were in another compartment, and for cystocele, about one third of retreatments were in another compartment. Prolapse in a new compartment should not be considered to be recurrence, in our opinion.

The high participation rate (> 70%) 5 years after operation was not expected. We believe the high response rate to be a consequence of the patients’ wishes to contribute and the patient-oriented validation of the questionnaire [23].

Following up PROMs through a questionnaire after 5 years proved to be feasible and reliable. During validation rounds, none of the previously used questions (that is, taken from the 1-year questionnaires) were criticized. This underlines the validity of the previously used questionnaires regarding quantifying and assessing patients’ real-life experiences with POP repair.

A possible weakness of the study is the exclusion of re-operated patients from the PROM survey after 5 years. However, since a substantial number of patients’ re-operations were in another compartment, this exclusion would not have reflected the results of an original POP procedure with native repair. To capture the most severe relapses, we instead chose to register all re-operations during an exceptionally long follow-up period.

A possible weakness of the questionnaire study could have been selection bias. However, there were no important differences between the responding patients and the excluded patients and the non-responders, nor was there a difference between questionnaires answered over e-mail or through regular mail. Therefore, any effect of selection bias on the results was probably minor at most.

Another possible selection bias was exclusion of patients without an e-mail address. The largest group lost to follow-up for this reason consisted of patients who were operated using native tissue. Among the patients we were able to reach, there was a slight mean age difference between patients followed up via letter and patients followed up via e-mail. However, no difference in response rates was found between these two groups, and therefore we concluded that an electronic survey is both economical and feasible even for a relatively elderly population.

The use of a non-surgically evaluated cure rate is a concern for many, especially due to misclassifications. De novo prolapse in a new compartment, for example, would be reported as a failed operation even though it might be unrelated to the original surgical procedure. This reporting procedure might overestimate the total amount of failure, but it probably would not influence the differences between groups. Additionally, because the patients’ wellbeing and self-reported lack of bulging symptoms are the goals of the operation, it is our belief that the anatomical evaluation is secondary. The value of PROMs compared with objective assessments is under constant debate. The medical indication for POP surgery is not the actual anatomical defect but rather the burden of subjective symptoms. POP is a condition which “requires no treatment when asymptomatic” [24]. Verification via POPQ (Pelvic Organ Prolapse Quantification System) would not alter a patient’s symptoms and cannot predict whether an operation would be successful or not from the patient’s perspective.

Retreatment of pelvic organ prolapse was defined solely as the patient receiving another surgical intervention. This definition means that minor issues and all treatments and results with pessaries, pelvic floor therapy, etc., were not assessed. The lack of subjective patient data underlines the value of backing up the Kaplan-Meier estimates with patient questionnaires to be able to assess the results more holistically .

Our PROM data showed that patients who had not had a re-treatment since their original operation 5 years previously reported that they were cured (i.e., no longer felt a vaginal bulge) in > 70% of cases. In addition, they had an equally high rate of patient satisfaction and symptom reduction overall. Therefore, combined with the re-operation rates, native tissue repair yielded satisfactory long-term results, which contrasts with other studies of the long-term effectiveness of native tissue repair [22, 25–27]. The conflicting results are probably due to the different outcome measures used. Nevertheless, it should be noted that more recent studies [28, 29] have also suggested that the native tissue repair approach could be better than previously thought.

PROMs gave us an overview of the patients’ various problems related to POP, including the effects of surgery on those symptoms. Some of our previous works based on GynOp data have shown that subjective symptoms are significantly reduced 1 year postoperativly [12, 14, 15], but in the present study we were able to show lasting results over 5 years. All these subjective symptoms were still significantly reduced compared with the preoperative situation. The only negative effect noted was that sexual activity was reduced (which was expected since the patients were 5 years older at follow-up [30]).

For this study, we chose not to analyse PROM data from the group operated for prolapse of the vaginal apex. This is a heterogeneous group with many varying factors regarding operative technique and prerequisite factors. In general, to use and compare PROM data in a clinically applicable fashion, it is preferable to use homogeneous groups with selected patients, as was done here. Recently, a large meta-analysis found that randomized controlled trials conducted on patients who had undergone prolapse surgery of the vaginal apex compartment had highly variable outcome measures and results, which underlines the suspected difficulties [2].

## Conclusions

- Native tissue repair of the most common types of POP is successful from a long-term perspective, as evidenced by our finding that re-operations were performed in 15% of cases after 10 years.
- A systematic follow-up by questionnaire is equally feasible via letter via e-mail even in a relatively older group of patients.
- Overall, PROMs show that native tissue repair yields satisfactory results after 5 years with a durable reduction in several core symptoms related to POP and a cure rate consistently > 70%.

## Supplementary information

ESM 1 (DOCX 76 kb)

ESM 2 (JPG 91 kb)

## Abbreviations

GynOp: National Quality Register for Gynaecological Surgery
POP: Pelvic organ prolapse
PROM: Patient-Reported Outcome Measurements
