# Supplementary material for: Long-term outcome after routine surgery for pelvic organ prolapse—A national register-based cohort study
Source: Int Urogynecol J. 2022 Mar 21;33(7):1863–73. doi: 10.1007/s00192-022-05156-y (PMC9270303; doi:10.1007/s00192-022-05156-y)
Supplement: Supplementary file 1 — (DOCX 76 kb) [file 192_2022_5156_MOESM1_ESM.docx]

| 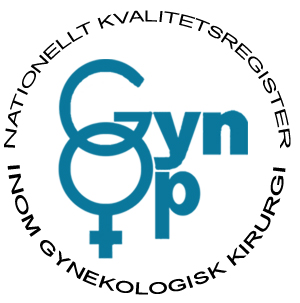 | Gynop-registret  Kvinnokliniken, Norrlands universitetssjukhus  901 85 Umeå  Telefon: 090-785 04 64  Telefax: 090-13 75 40  E-post: <mailto:>Hemsida: [www.gynop.org](http://www.gynop.org) | 2019-08-20 | Sida 1 (6) - 1 - - 1 -Sidan 1 av 1Sidan 1 av 1 |
| --- | --- | --- | --- |

Påminnelse

Bästa

Du opererades vid XXXXXXXXX kvinnoklinik för framfall [datum] .

Vi önskar din hjälp för att öka vår kunskap om långtidsresultat efter den operationen.

Din kvinnoklinik är vidtalad och har godkänt att vi kontaktar dig.

Din enkät finns även på www.gynop.se, klicka "Logga In". Ditt lösenord är «PatientID».

Självfallet är deltagandet frivilligt

Har du frågor angående denna enkät eller studie kontakta:

Emil Nüssler, Överläkare emeritus

Registeransvarig för framfallskirurgi inom

Nationella kvalitetsregistret inom gynekologisk kirurgi

Norrlands universitetssjukhus

901 85 Umeå

Emil.Nussler@umu.se

| Din värdering efter operationen  **(ca 5 år)** |  |  |
| --- | --- | --- |
| Personnummer: ...... ....... ....... - ....................  Namn...............................................................  Adress..............................................................  Postnummer.............Ort...................................  Tel. bost ....................... Tel. arb......................  Övrig telefon ...................................  E-post...................................................…......... |  | Gult markerat frågor ur befintliga enkäter. Ej markerat nya frågor för femårsenkäten |

**Detta är en uppföljning av den framfallsoperation du genomgick för 5 år sedan.**

Vi undrar hur du har det idag.

1. Datum när enkäten ifylls:………………………………………………………..

2. Har du en känsla av att något buktar ut ur slidan?

🞏 Aldrig 🞏 Nästan aldrig 🞏 1–3 ggr per månad 🞏 1–3 ggr per vecka 🞏 Dagligen

3. Har du skavningsbesvär i underlivet?

🞏 Aldrig 🞏 Nästan aldrig 🞏 1–3 ggr per månad 🞏 1–3 ggr per vecka 🞏 Dagligen

4. Har du svårt att tömma urinblåsan?

🞏 Aldrig 🞏 Nästan aldrig 🞏 1–3 ggr per månad 🞏 1–3 ggr per vecka 🞏 Dagligen

5. Har du besvär av urinträngningar (en hastigt påkommande, stark känsla av att behöva kissa)?

🞏 Aldrig 🞏 Nästan aldrig 🞏 1–3 ggr per månad 🞏 1–3 ggr per vecka 🞏 Dagligen

6. Behöver du gå upp på natten och kissa?

🞏 Aldrig 🞏 Enstaka nätter 🞏 Oftast en gång 🞏 Oftast två ggr 🞏 Mer än två ggr

7. Har du urinläckage eller ofrivillig urinavgång?

🞏 Aldrig 🞏 Nästan aldrig 🞏 1–3 ggr per månad 🞏 1–3 ggr per vecka 🞏 Dagligen

8. Händer det att du har svårt att tömma tarmen?

🞏 Aldrig 🞏 Nästan aldrig 🞏 1–3 ggr per månad 🞏 1–3 ggr per vecka 🞏 Dagligen

9. Händer det att du behöver hålla emot bakre slidväggen för att tömma tarmen?

🞏 Aldrig 🞏 Nästan aldrig 🞏 1–3 ggr per månad 🞏 1–3 ggr per vecka 🞏 Dagligen

10. Har du läckage av fast avföring?

🞏 Aldrig 🞏 Nästan aldrig 🞏 1–3 ggr per månad 🞏 1–3 ggr per vecka 🞏 Dagligen

11a. Har din läkare konstaterat ett nytt framfall (prolaps)
 efter operationen för fem år sedan?

🞏 Nej

🞏 Ja

🞏 Vet inte

🞏 Ingen läkarundersökning utförd

11b. Om ja, har du blivit opererad för det nya framfallet?

🞏Ja 🞏 Nej

11c. Tror du själv att du har ett nytt framfall nu?

🞏 Ja 🞏 Nej

12a.Lades det in ett nät vid operationen för fem år sedan?

🞏 Ja 🞏Nej 🞏Vet ej

[Underfrågarna 12 b 13b öppnas endast om svaret är Ja]

12b. Om ja på fråga 12 a, Har du haft några problem som du tror beror på nätet?

🞏 Nej

🞏 Ja, framfallet kom tillbaka

🞏 Ja, blödning eller flytningar

🞏 Ja, en känsla av att slidan har blivit för trång

🞏 Ja, smärtor/värk

🞏 Ja, smärtor/obehag hos min partner vid samlag

🞏 Annat……………………………

12c. Om ja på fråga 12 a, har du och/eller din läkare konstaterat att:

🞏 Nätet tränger igenom slidväggen

🞏 Nätet tränger in i urinblåsan

🞏 Nätet tränger in i ändtarmen

🞏 Det har uppstått inflammation/infektion omkring nätet

🞏 Annat……………………………

🞏 Vet ej

13a. Har du blivit opererad igen på grund av nätet?

🞏 Ja 🞏 Nej

[Underfrågan 13b öppnas endast Om JA på fråga 13a]

13b. Om ja på fråga 13 a,

🞏 Delar av nätet har opererats bort

🞏 Hela nätet har opererats bort

🞏 Annan operation pga nätet ……………………………

14a Har du haft samlag de senaste 3 månaderna?

🞏 Ja 🞏 Nej [fråga 14b +14 c+ 14d öppnas endast om svar Ja på fråga 14a]

14b. Om ja på fråga 14 a: Känner du smärta i underlivet vid samlag?

🞏 Nej, ingen smärta

🞏 Ja, lite smärta

🞏 Ja, måttlig smärta

🞏 Ja, stark smärta

🞏 Ja, olidlig smärta

14c. Om ja på fråga 14 a: Upplever du

slidöppningen alltför liten/trång? 🞏 Ja 🞏 Nej

slidöppningen alltför stor/öppen? 🞏 Ja 🞏 Nej

smärta i slidöppningen? 🞏 Ja 🞏 Nej

andra besvär från slidöppningen? 🞏 Ja 🞏 Nej

[underfrågan ”Om ja, vilken typ av besvär” öppnas endast om svar Ja på frågan om ”andra besvär från slidöppningen]

Om ja, vilken typ av besvär? …………………………………………………………….

……………………………………………………………………………………………

14d. Om ja på fråga 14 a: Har din partner påtalat återkommande besvär vid samlag som ni relaterar till framfallsoperationen?

🞏 Ja 🞏 Nej

[underfrågan ”beskriv besvären” öppnas endast om svar Ja på frågan 14a]

Om ja, beskriv besvären: ………………………………………………………………………..

……………………………………………………………………………………………

**Det finns några andra allmänna faktorer som kan påverka resultatet som vi även önskar svar på**

15a. Har du haft menstruationer/underlivsblödningar under det senaste året?

🞏 Ja 🞏 Nej[Bort: If ålder>=60] [underfrågorna 15b-d öppnas endast om svar JA på frågan 15]

15b. Kommer dina menstruationsblödningar med jämna intervall? 🞏 Ja 🞏 Nej

15c. Har du blödningar som kommer när de inte borde komma? 🞏 Ja 🞏 Nej

15d. Äter du hormonpreparat som ger förväntade blödningar? 🞏 Ja 🞏 Nej

16. Använder du hormonpreparat med östrogen?
[Bokmärke: XHormonpreparat, Bort 17a-b: If ålder<=40]

🞏 Nej

🞏 Ja, mot övergångsbesvär/klimakteriebesvär

🞏 Ja, mot underlivsproblem

🞏 Ja, mot problem med urin/urinvägar

🞏 Ja, som preventivmedel [Villkor: svarsalternativ om >=40 och <=50]

🞏 Ja, av annan anledning .............................................................................

17a. Hur lång är du? ......... cm b. Hur mycket väger du? ........ kg

18. Röker du?

🞏 Ja, ungefär…….(antal) cigaretter per dag

🞏 Nej, slutade år ................

🞏 Nej, har aldrig rökt

19. Har du sedan framfallsoperationen [datum] medicinerat regelbundet (dagligen eller varje vecka)
med kortison (tabletter eller sprutor, dock inte kräm/salva) t ex Prednisolon, Prednison, Betapred, Betametason, Dexametason, Kortisonacetat?

🞏 Nej, aldrig
🞏 Ja men mindre än 1 år
🞏 Ja, ungefär ………..år( accepterar heltal 1 till 5 – funktion inget som syns)
🞏 Ja, i princip hela tiden

**Till sist önskar vi ställa några övergripande frågor om framfallsoperationen**

20. Vad anser du om resultatet av framfallsoperationen som utfördes för 5 år sedan? Mitt tillstånd är:

🞏 Mycket förbättrat
🞏 Förbättrat
🞏 Oförändrat
🞏 Försämrat
🞏 Mycket försämrat

21. Vad tycker du om resultatet av framfallsoperationen som utfördes för 5 år sedan?

🞏 Mycket nöjd
🞏 Nöjd
🞏 Varken nöjd eller missnöjd
🞏 Missnöjd
🞏 Mycket missnöjd

22. Du som angivit komplikationer eller operationer på någon av frågorna, godkänner du att vi får
ta del av journalhandlingar om detta
🞏 Nej 🞏 Ja

23. Finns det något ytterligare som hänger samman med framfallsoperationen
som du vill berätta om

....................................................................................................................................................................

....................................................................................................................................................................

....................................................................................................................................................................

24. Har du haft problem med att förstå någon eller några frågor i enkäten?

🞏 Nej 🞏 Ja

[underfrågan öppnas endast om svar Ja på frågan 24]

Om ja, skriv numret på frågan och beskriv problemet:

.........................................................................................................................................................

.........................................................................................................................................................

.........................................................................................................................................................

*Namn (den som fyllt i formuläret)*
