# Supplementary figures and images for: Long-term outcome after routine surgery for pelvic organ prolapse—A national register-based cohort study
Source: Int Urogynecol J. 2022 Mar 21;33(7):1863–73. doi: 10.1007/s00192-022-05156-y (PMC9270303; doi:10.1007/s00192-022-05156-y)

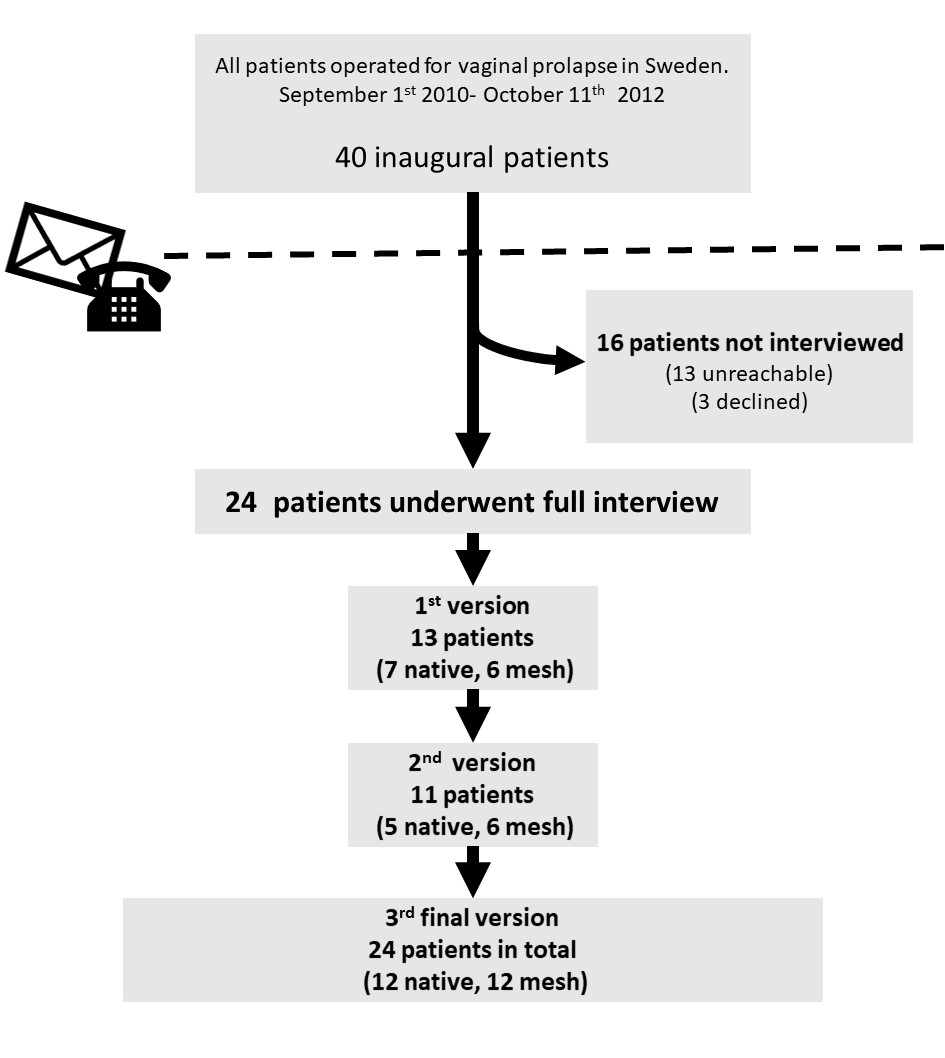

Supplement: Supplementary file 2 — (JPG 91 kb) [file 192_2022_5156_MOESM2_ESM.jpg]
